# The Real-World Impact of Obesity on Quality of Life and Work Productivity in Spain: A Cross-Sectional Observational Study Involving Physicians and Individuals With Obesity

**DOI:** 10.7759/cureus.105867

**Published:** 2026-03-26

**Authors:** Gemma Rodríguez-Carnero, Jennifer Redondo-Antón, Silvia Díaz-Cerezo, Ashley Mortimer, Tamara Mensah, Esther Artime, Miriam Rubio-de Santos

**Affiliations:** 1 Endocrinology and Nutrition Service, University Hospital Complex of Santiago de Compostela, Santiago de Compostela, ESP; 2 Epigenomics in Endocrinology and Nutrition Group, Epigenomics Unit, Health Research Institute of Santiago de Compostela (IDIS), Santiago de Compostela, ESP; 3 Medical Department, Eli Lilly and Company, Indianapolis, USA; 4 Cardiovascular Renal Metabolic Liver (CVRML) Franchise, Adelphi Real World, Bollington, GBR

**Keywords:** body mass index, health-related quality of life, obesity, overweight, work productivity

## Abstract

Background: Understanding how overweight and obesity impact quality of life, psychological well-being, and social functioning is essential for informing and optimizing patient-centric disease management strategies.

Aims: This study aims to investigate the humanistic burden of excess body weight in adults with overweight or obesity enrolled in weight management programs under routine clinical practice settings in Spain, including emotional well-being, health-related quality of life, and work productivity and activity impairment, the latter assessed via validated patient-reported outcome instruments (36-Item Short Form Health Survey version 2 (SF-36v2) and Work Productivity and Activity Impairment (WPAI)).

Methods: The study uses data from the multinational Adelphi Obesity Disease Specific Programme™, a cross-sectional survey with retrospective data collection conducted in Spain (November 2023 to March 2024). Adults with a prior/current diagnosis of obesity (BMI ≥30 kg/m²) or overweight (BMI ≥27 kg/m²) with ≥1 obesity-related comorbidity, enrolled in a weight management program, were eligible. Physicians collected clinical data from the medical records of their first eight eligible patients, who were invited to complete a voluntary questionnaire including emotional impact and patient-reported outcome measures: SF-36v2 (T-scores <47 = impaired functioning), evaluating health-related quality of life (HRQoL), and WPAI (0 = no effect; 100% = completely prevented activity). Descriptive analyses were performed overall and by BMI and Edmonton Obesity Staging System-defined subgroups.

Results: A total of 106 physicians reported data from 801 eligible adults, of whom 330 (41.2%) provided self-reported data; 88.3% (n = 707) presented ≥1 obesity-related disease, commonly hypertension (41.2%, n = 330), dyslipidemia (32.8%, n = 263), and anxiety (18.9%, n = 151); 77.4% (n = 620) were on supervised diet, and 58.6% (n = 469) followed guided exercise programs. 54.2% (n=195) experienced weight loss followed by regain. Difficulties maintaining diet and exercise programs (58.8%, n=190) and insufficient weight loss (50.2%, n=162) were the main reasons cited for discontinuing weight loss efforts. Mean SF-36v2 scores were <47 for all domains except vitality, indicating impairment across multiple dimensions. On average, participants reported that 18.8% of their work productivity and 29.7% of their daily activities were impaired due to overweight/obesity.

Conclusion: In Spain, analysis suggests that overweight and obesity may be associated with lower HRQoL and decreased levels of daily and work productivity.

## Introduction

Excess body fat accumulation, leading to both overweight and obesity, constitutes a major global public health concern due to its high prevalence and its well-documented clinical, economic, and societal burden [1]. The prevalence of this chronic, relapsing, and multifactorial disease has increased over the past few decades [2-4], and it is expected to continue growing. In Spain, the most recent estimates indicate that 56% of the adult population lives with overweight, while 19% lives with obesity [5]. Projections indicate that by 2035, the prevalence of obesity will nearly double, affecting 37% of Spanish adults [6,7].

The World Health Organization defines health as a complete state of physical, mental, and social well-being [8], underscoring the disabling burden of obesity, which contributes not only to deterioration in health but also to psychological distress and social exclusion, thereby reducing overall quality of life [9]. Excess body fat increases the risk of developing obesity-related diseases (ORD), including biomechanical and metabolic conditions [1], leading to reduced life expectancy [10]. Additionally, overweight and obesity are linked to substantial psychological effects, often exacerbated by weight bias, which stems from assumptions of personal responsibility [11]. Individuals with obesity report higher rates of psychiatric disorders, such as depression and anxiety, and experience lower self-esteem and poorer self-rated health, compared with those without obesity [12]. As a result of excess body weight, people often encounter discrimination, prejudice, and negative attitudes, adversely affecting their mood, self-image, and even their commitment to dietary-behavioral treatment [13].

The complex and chronic nature of overweight and obesity has been postulated as having significant economic consequences for both individuals and countries. Obesity is often linked to higher direct healthcare costs, mostly associated with treating ORD. In accordance with this, individuals with obesity tend to use more healthcare services than those without obesity [14]. However, direct healthcare costs represent only 32% of the total obesity-related expenditure, with the remaining 68% arising from indirect costs secondary to decreased productivity, work absenteeism, and total disability [15]. Studies have shown that people with obesity miss more days at work than those without obesity and are more likely to report poor work capability or limitations in the amount, type, or quality of work conducted [14,16,17]. Despite the significant burden of obesity, its management remains inadequately prioritized in clinical settings [18]. Although progress has been made in recognizing obesity as a chronic disease and its pathophysiology, misconceptions about its treatment persist, leading to gaps in its management [3]. Current clinical guidelines recommend a holistic and individualized approach to obesity management, incorporating healthy eating habits, physical activity, behavioral therapy, pharmacological treatment, endoscopic options, and bariatric surgery, along with specialized psychological support when needed [11,19]. This framework supports tailored treatment plans to each person’s individual needs and preferences and implementation of these through multidisciplinary healthcare professional (HCP) teams [11]. Obesity treatment has demonstrated beneficial effects on cardiometabolic parameters [20] and quality of life outcomes [21].

To date, no studies have thoroughly examined the real-world emotional impact of excess body weight or how overweight and obesity influence health-related quality of life (HRQoL) and work productivity and activity outcomes within routine clinical practice settings in Spain. Understanding these aspects is essential for developing person-centered, holistic approaches that enhance not only physical health but also emotional and social well-being. Ultimately, this knowledge can help drive more inclusive healthcare policies and interventions. The present study explored the humanistic burden of excess body weight among people living with overweight and obesity (hereafter referred to as PwO) enrolled in weight management programs in Spanish real-world clinical practice settings, according to disease severity.

## Materials and methods

Study design

This secondary analysis utilized data from the Adelphi Real World Obesity Disease Specific Program (DSP)™, a cross-sectional survey with retrospective data collection conducted in Spain between November 2023 and March 2024.

The DSPs are structured real-world evidence generation initiatives developed and managed by Adelphi Real World, aimed at gathering information across multiple therapeutic areas through surveys targeted at physicians and patients. DSPs are not designed to address a predefined research hypothesis but to generate a broad body of evidence that serves as an objective, impartial data source to support diverse analyses of healthcare delivery and patient-centered outcomes. The methodological framework underlying DSPs has been previously described [22,23] and validated [24] in the field of health outcomes research and demonstrated to be both representative and consistent over time [25].

For this study, relevant variables were extracted from the Obesity DSP dataset to address the specific research objectives. Data from the Spanish sample are presented here.

Study participants

The DSP study population in Spain comprised primary care physicians (PCPs), internists, diabetologists, and endocrinologists, along with their consulting PwO. Participating physicians were recruited through local fieldwork partners and were eligible for inclusion if they were personally responsible for the management and treatment decisions for PwO, saw a minimum of 10 PwO in a typical month, and accepted all survey requirements.

Included PwO were ≥18 years at the time of data capture, on a weight management program, and had a physician-confirmed diagnosis based on a previous or current (at data collection) BMI ≥30 kg/m² or a previous or current BMI ≥27-<30 kg/m² with at least one ORD. Since PwO could be included based on a prior overweight or obesity diagnosis, some PwO may present with a BMI <30 kg/m² at data capture, indicating some PwO may have achieved their weight loss goals since diagnosis.

Table 1 presents the inclusion and exclusion criteria.

**Table 1:** Selection criteria for DSP participation * People with BMI ≥30 kg/m² at the time of data collection may have achieved weight loss and/or BMI ≥27 kg/m² with at least one obesity-related complication. Abbreviations: PwO: people living with overweight and obesity; BMI: body mass index; DSP: Disease Specific Program.

Healthcare professionals	PwO
Specialists in Family and Community Medicine, Endocrinology, or Internal Medicine	Adults 18 years of age or older on a weight management program
Responsible for the management and treatment decisions of people with obesity	Current (data collection) or previous physician-confirmed diagnosis of obesity*: BMI ≥30 kg/m², BMI ≥27 to <30 kg/m^2^ in the presence of at least one complication associated with obesity
Serving a minimum of 10 people with obesity in a typical month	Not participating in an obesity clinical trial at the time of data collection

PwO were further classified according to treatment with an obesity management medication (OMM) at the time of data collection (yes/no).

Physician- and patient-reported questionnaires

As part of the DSP, eligible physicians were instructed to complete a questionnaire for the first eight PwO they consulted who met the inclusion criteria. This form captured clinical and obesity-related characteristics, as well as physician perceptions of their PwO.

To ensure sufficient representation of PwO using OMM, at least two out of every eight were required to be on OMM. Consequently, PwO were categorized into two groups: (1) PwO receiving OMM and (2) PwO not receiving OMM.

PwO, whose physicians completed a questionnaire, were asked to complete a voluntary and confidential questionnaire immediately following their consultation. This questionnaire included general items to assess the emotional impact of obesity, along with validated patient-reported outcome measures (PROMs) to evaluate HRQoL and limitations in work performance and daily functioning, using the 36-Item Short Form Health Survey version 2 (SF-36v2) [26,27] and the Work Productivity and Activity Impairment (WPAI) questionnaire [28], respectively.

The questionnaire was part of Adelphi Real World's DSP methodology [22,23], which was designed to capture overall experiences of individuals living with overweight or obesity. For this study, the variables relevant to our specific objective of assessing emotional impact were extracted, as detailed in the study measures.

Study measures

The measures analyzed in our study are outlined below.

Clinical and Obesity Characteristics

Clinical characteristics collected for PwO included sex, age, and concomitant medication use. Obesity-specific data encompassed body weight and BMI at data capture, the interval between diagnosis and data capture, and BMI at the time of diagnosis. The Edmonton Obesity Staging System (EOSS) [29] stage (Stage 1 = pre-clinical risk factors, 2 = established complications, 3 = end-organ damage, 4 = end-stage) (Supplemental Material 1) and the presence of ORDs were also documented.

Obesity Management

Concerning the weight loss journey, data collected included current and previous interventions for obesity management and the change in BMI from diagnosis to data capture. Weight loss trajectories were described using the following categories: never lost weight, lost and regained weight, and lost and maintained weight.

Emotional Impact of Obesity

Perspectives of PwO regarding challenges in weight loss, satisfaction with treatment approaches, the emotional and psychosocial impacts of obesity, and their objectives and weight-loss goals were assessed.

HRQoL Impact of Obesity

HRQoL was assessed using the SF-36v2 [26], which evaluates eight health domains: physical functioning, bodily pain, role limitations due to physical health problems (role-physical), role limitations due to emotional problems (role-emotional), emotional well-being (mental health), social functioning, energy/fatigue (vitality), and general health perceptions. From these domains, two overall summary scores were calculated: the Physical Component Summary (PCS) and the Mental Component Summary (MCS). T-scores, which assess deviation from the mean, were interpreted based on normative data from the 2009 U.S. general population (mean ± standard deviation (SD): 50 ± 10). A T-score between 47 and 53 was considered within the normal range, while scores below 47 indicated impairment in the respective domain [27]. Use of the SF-36 questionnaire was conducted under the appropriate license from QualityMetric Incorporated (License: QUO-01306-K8K3G3) for the purposes of this study.

Impact of Obesity on Work-Related Outcomes

Employment status at data capture was reported. Obesity-related productivity loss and activity impairment were evaluated using the validated WPAI questionnaire. This tool measures absenteeism (percentage of work hours missed due to obesity), presenteeism (percentage of impairment while working due to obesity), and overall work impairment (total percentage of work hours affected by obesity) among currently employed PwO. Additionally, it assesses overall activity impairment (the percentage of daily activities affected by obesity) for all PwO, regardless of employment status. WPAI scores range from 0% to 100%, with higher scores indicating greater impairment [28].

Data analysis

Since the study objectives were purely descriptive, the sample size was determined by the DSP data collection methodology, which only affected the precision of the estimates. All subjects meeting the eligibility criteria were included in this secondary analysis.

As the Adelphi DSP methodology relies on voluntary physician participation sampling rather than random selection, the probability of inclusion is unknown for both physicians and people with obesity. As a result, adjustments to correct sampling imbalances are not applicable. The study is descriptive in nature and was not designed to produce population‑level estimates but to characterize patterns within the sampled population. Although some comparisons were made, they were exploratory rather than hypothesis-driven, aiming to examine subgroup differences in weight status and disease characteristics.

Clinical data recorded by HCPs were matched, when available, to the corresponding patient-reported information. As completion of the patient questionnaire was voluntary, not all physician-reported cases have associated patient input. Analyses involving patient-reported data were conducted using the matched dataset.

Participants were categorized according to their BMI at the time of data collection: normal weight and overweight (BMI <30 kg/m²), Class I obesity (BMI ≥30 and <35 kg/m²), Class II obesity (BMI ≥35 and <40 kg/m²), and Class III obesity (BMI ≥40 kg/m²).

Descriptive analyses were performed on the total sample and within BMI- and EOSS-based subgroups. Categorical variables were summarized using relative frequencies and percentages, while numerical variables were reported as mean, SD, median, 25th and 75th percentiles, and minimum and maximum values. Missing data were not imputed; therefore, the base for analysis could vary from variable to variable and is reported separately for each analysis. All analyses were performed using UNICOM Intelligence Reporter, Version 7.5 (Released 2021; UNICOM Systems, Inc., Mission Hills, California).

Validated PROMs were scored following the official user manual and scoring guidelines provided by the instrument's developer, in compliance with copyright regulations.

## Results

Overall, data from 106 physicians (49 (46%) endocrinologists/diabetologists, 47 (44%) PCPs, and 10 (9%) internists) and 801 PwO were analyzed, of whom 174 (21.7%) were receiving OMM and 627 (78.3%) were not. Additionally, 330 (41.2%) PwO voluntarily completed the self-reported questionnaire.

Clinical and obesity characteristics

Among all included PwO, 63.8% (n = 511) were female, with a mean ± SD age of 51.0 ± 13.7 years and a BMI of 35.4 ± 6.5 kg/m² at the time of data collection (Table 2). The mean ± SD duration from obesity diagnosis to data collection was 5.0 ± 6.3 years. At the time of diagnosis, PwO had a mean BMI of 37.4 ± 6.8 kg/m²; 84.4% (n = 676) of PwO exhibited some degree of obesity-related health impairment (EOSS ≥1). On average, PwO had 2.5 ± 2.2 ORD, with 88.3% (n=707) presenting with at least one. Individuals in Class II or III obesity had a higher mean number of ORD (2.9 ± 2.5 for Class II and 2.9 ± 2.3 for Class III) and a higher proportion of PwO with at least one (92.7% (n=204) and 92.9% (n=144), respectively). Overall, the most frequently reported complications were hypertension (41.2%, n=330), dyslipidemia (32.8%, n=263), and anxiety (18.9%, n=151). Demographic and clinical characteristics of PwO by EOSS categories are presented in the Appendices (Supplemental Material 2).

**Table 2:** Demographic and clinical characteristics of PwO at the time of the survey by BMI at data collection ^ Includes “full-time homemaker,” “student,” “unemployed,” and “on long-term sick” categories. ^^ Includes “completed mandatory education,” “did not complete mandatory education,” and “other.” * Edmonton Obesity Staging System, calculated based on EOSS criteria using variables available within the DSP (Supplemental Material 1). ** Those present in >10% of participants were included. Abbreviations: ACE, angiotensin-converting enzyme; ARB, angiotensin-II receptor; BMI, body mass index; EOSS, Edmonton Obesity Staging System; PwO, people with overweight/obesity; SD, standard deviation; SGLT-2, sodium-glucose cotransporter 2; T2DM, type 2 diabetes mellitus.

Variable	Total n=801 (100%)	BMI<30 n=135 (16.9%)	30≥BMI <35 n=291 (36.3%)	35≥BMI <40 n=220 (27.5%)	BMI≥40 n=155 (19.4%)
Age, mean (SD)	51.0 (13.7)	52.4 (13.6)	51.9 (13.7)	51.2 (14.2)	47.9 (12.9)
Sex, women; n (%)	511 (63.8)	87 (64.4)	173 (59.5)	141 (64.1)	110 (71.0)
BMI at diagnosis, mean (SD)	37.4 (6.8)	31.4 (3.2)	34.8 (4.1)	39.2 (3.9)	46.0 (7.8)
BMI at data collection; mean (SD)	35.4 (6.5)	27.6 (1.9)	32.5 (1.4)	37.2 (1.5)	45.3 (6.0)
Time from diagnosis, years; mean (SD)	5.0 (6.3)	3.7 (4.5)	4.6 (6.5)	5.5 (6.9)	6.4 (6.4)
Smoking status, n	778	133	286	211	148
Never smoked, n (%)	462 (59.4)	86 (64.7)	159 (55.6)	124 (58.8)	93 (62.8)
Ex-smoker, n (%)	201 (25.8)	37 (27.8)	79 (27.6)	54 (25.6)	31 (21.0)
Current smoker, n (%)	115 (14.8)	10 (7.5)	48 (16.8)	33 (15.6)	24 (16.2)
Employment status, n	320	58	123	91	48
Working, n (%)	196 (61.3)	38 (65.5)	80 (65.0)	49 (53.8)	29 (60.4)
Not working^, n (%)	60 (18.8)	8 (13.8)	20 (16.3)	20 (22.0)	12 (25.0)
Retired, n (%)	64 (20.0)	12 (20.7)	23 (18.7)	22 (24.2)	7 (14.6)
Educational status, n	327	59	127	89	52
University degree or higher	114 (34.9)	33 (55.9)	46 (36.2)	24 (27.0)	11 (21.1)
Completed non-mandatory secondary education	60 (18.3)	9 (15.3)	22 (17.3)	14 (15.7)	15 (28.8)
Mandatory education or lower^^	153 (46.8)	17 (28.8)	59 (46.5)	51 (57.3)	26 (50.0)
EOSS Category*; n (%)					
EOSS 0	125 (15.6)	34 (25.2)	48 (16.5)	24 (10.9)	19 (12.3)
EOSS 1–2	392 (48.9)	69 (51.1)	147 (50.5)	105 (47.7)	71 (45.8)
EOSS 3–4	284 (35.5)	32 (23.7)	96 (33.0)	91 (41.4)	65 (41.9)
Obesity-related diseases (ORD), mean (SD)	2.5 (2.2)	2.1 (1.9)	2.3 (1.9)	2.9 (2.5)	2.9 (2.3)
Obesity-related diseases, n (%)**					
Any obesity-related disease	707 (88.3)	113 (83.7)	246 (84.5)	204 (92.7)	144 (92.9)
Hypertension	330 (41.2)	48 (35.6)	113 (38.8)	110 (50.0)	59 (38.1)
Dyslipidemia	263 (32.8)	44 (32.6)	81 (27.8)	83 (37.7)	55 (35.5)
Anxiety	151 (18.9)	21 (15.6)	51 (17.5)	41 (18.6)	38 (24.5)
T2DM without chronic complications	112 (14.0)	24 (17.8)	30 (10.3)	42 (19.1)	16 (10.3)
Depression	110 (13.7)	15 (11.1)	35 (12.0)	37 (16.8)	23 (14.8)
Osteoarthritis	99 (12.4)	14 (10.4)	36 (12.4)	24 (10.9)	25 (16.1)
Lumbago	96 (12.0)	15 (11.1)	36 (12.4)	23 (10.5)	22 (14.2)
Hypothyroidism	90 (11.2)	19 (14.1)	27 (9.3)	26 (11.8)	18 (11.6)
Concomitant medication use, n	707	113	246	204	144
Concomitant medications**, n (%)					
Any concomitant medication	658 (93.1)	107 (94.7)	228 (92.7)	190 (93.1)	133 (92.4)
Statin	274 (38.8)	37 (32.7)	96 (39.0)	90 (44.1)	51 (35.4)
ARB inhibitors	202 (28.6)	26 (23.0)	74 (30.1)	59 (28.9)	43 (29.9)
Anti-depressants	172 (24.3)	19 (16.8)	51 (20.7)	61 (29.9)	41 (28.5)
Metformin/Biguanide	155 (21.9)	25 (22.1)	53 (21.5)	52 (25.5)	25 (17.4)
Benzodiazepines	148 (20.9)	26 (23.0)	57 (23.2)	38 (18.6)	27 (18.8)
ACE inhibitors	119 (16.8)	23 (20.4)	30 (12.2)	48 (23.5)	18 (12.5)
Pain medication	112 (15.8)	24 (21.2)	37 (15.0)	29 (14.2)	22 (15.3)
Other diuretics	92 (13.0)	13 (11.5)	22 (8.9)	35 (17.2)	22 (15.3)
SGLT-2 inhibitors	86 (12.2)	18 (15.9)	26 (10.6)	32 (15.7)	10 (6.9)
Calcium channel blockers	84 (11.9)	7 (6.2)	29 (11.8)	31 (15.2)	17 (11.8)

Obesity management

At the time of data capture, the primary interventions for obesity management consisted of diet and exercise that were either supervised/agreed upon with an HCP. Only 10.4% (n=83) of participants had access to professional behavioral therapy as part of their treatment plan. Prior to the current weight loss interventions, most PwO had attempted to manage overweight/obesity through their own diet or exercise routines (Table 3).

**Table 3:** Description of obesity management interventions and weight loss journey *Values with N≤3 have been suppressed to protect confidentiality. Additional cells may also have been suppressed to prevent the deduction of small counts. The 10 most frequent options have been selected. Abbreviations: BMI, body mass index; HCP, health care professional; PwO, people with overweight/obesity.

			Total; n=801 (100%)	BMI<30; n=135 (16.9%)	30≥BMI<35; n=291 (36.3%)	35≥BMI<40; n=220 (27.5%)	BMI≥40; n=155 (19.4%)
Previous interventions PwO has tried, n (%)							
N available			781	134	284	213	150
Lifestyle changes	Diet	Patient’s own diet	584 (74.8)	106 (79.1)	214 (75.4)	151 (70.9)	113 (75.3)
		Diet recommended and supervised by an HCP	288 (36.9)	34 (25.4)	113 (39.8)	79 (37.1)	62 (41.3)
		Diet recommended and supervised by a dietitian	173 (22.2)	25 (18.7)	69 (24.3)	45 (21.1)	34 (22.7)
		Following a low-carb diet	139 (17.8)	27 (20.1)	53 (18.7)	30 (14.1)	29 (19.3)
		Following a Mediterranean diet	123 (15.7)	20 (14.9)	43 (15.1)	31 (14.6)	29 (19.3)
		Non-prescription diet foods	112 (14.3)	16 (11.9)	41 (14.4)	30 (14.1)	25 (16.7)
	Exercise	Patient’s own exercise regimen	410 (52.5)	72 (53.7)	149 (52.5)	116 (54.5)	73 (48.7)
		Exercise regimen agreed with HCP	116 (14.9)	11 (8.2)	55 (19.4)	30 (14.1)	20 (13.3)
	Behavioral therapy		40 (5.1)	6 (4.5)	15 (5.3)	10 (4.7)	9 (6.0)
Weight loss drugs			70 (9.0)	10 (7.5)	16 (5.6)	25 (11.7)	19 (12.7)
Weight loss surgery			22 (2.8)	… (*)	5 (1.8)	… (*)	11 (7.3)
Current interventions PwO is trying, n (%)							
N available			801	135	291	220	155
Lifestyle changes	Diet	Patient’s own diet	83 (10.4)	16 (11.9)	35 (12.0)	19 (8.6)	13 (8.4)
		Diet recommended and supervised by an HCP	620 (77.4)	103 (76.3)	238 (81.8)	166 (75.5)	113 (72.9)
		Diet recommended and supervised by a dietitian	223 (27.8)	29 (21.5)	71 (24.4)	72 (32.7)	51 (32.9)
		Following a low-carb diet	105 (13.1)	18 (13.3)	42 (14.4)	26 (11.8)	19 (12.3)
		Following a Mediterranean diet	230 (28.7)	42 (31.1)	86 (29.6)	58 (26.4)	44 (28.4)
		Non-prescription diet foods	17 (2.1)	… (*)	… (*)	… (*)	6 (3.9)
	Exercise	Patient’s own exercise regimen	215 (26.8)	35 (25.9)	78 (26.8)	68 (30.9)	34 (21.9)
		Exercise regimen agreed with HCP	382 (47.7)	50 (37.0)	155 (53.3)	103 (46.8)	74 (47.7)
		Exercise regimen agreed by a personal trainer	87 (10.9)	28 (20.7)	32 (11.0)	19 (8.6)	8 (5.2)
	Behavioral therapy		83 (10.4)	8 (5.9)	27 (9.3)	27 (12.3)	21 (13.5)
Weight loss drugs			174 (21.7)	29 (21.5)	52 (17.9)	51 (23.2)	42 (27.1)
Weight loss surgery			28 (3.5)	… (*)	… (*)	8 (3.6)	15 (9.7)
Evolution of weight loss; n (%)							
N available			360	57	130	103	70
Never lost weight			38 (10.6)	… (*)	… (*)	11 (10.7)	15 (21.4)
Weight loss followed by weight gain			195 (54.2)	22 (38.6)	74 (56.9)	62 (60.2)	37 (52.9)
Weight reduction and maintenance to date			127 (35.3)	32 (56.1)	47 (36.2)	30 (29.1)	18 (25.7)

Of all participants in the study, 407 (50.8%) were not considered candidates for weight loss surgery. The main reasons for non-eligibility were that participants either had a weight or risk assessment below the guideline recommendations for weight-loss surgery (n=189, 46.4%) or had not yet fully explored non-surgical treatment options (n=174, 42.8%). A total of 211 (26.3%) were identified as candidates, and 196 (31.0%) were eligible but had not received the procedure. At the time of data collection, 22 (2.8%) individuals had undergone weight loss surgery, and 28 (3.5%) were in the process of receiving it. Roux-en-Y gastric bypass (n=14, 30.4%) and sleeve gastrectomy (n=10, 21.7%) were the most common procedures (Supplemental Material 3).

Over a period of 5.0 ± 6.3 years from diagnosis to data capture, the mean BMI change was -2.2 ± 4.1 kg/m² (n=681). According to physician assessment, 54.2% (n=195) of PwO had experienced a pattern of initial weight loss followed by subsequent weight regain. Furthermore, 10.6% (n=38) of PwO had never been successful in losing any weight. The highest rate of unsuccessful weight loss was described in individuals with a BMI≥40 (Table 3).

Emotional impact of obesity and overweight

Primary motivations for weight loss among PwO included improving physical appearance, enhancing overall well-being, and fitting into smaller clothing. Over the past three years, PwO reported a mean of 5.0 ± 10.0 weight loss attempts, with 74.2% (n=239) failing to achieve their weight loss goal. A significant proportion (64.7%, n=209) reported having made either moderate (36.8%, n=119) or substantial (27.9%, n=90) lifestyle changes in their efforts to manage weight. However, despite doing their best to follow diet/exercise plans, 72.4% (n=231) reported lack of success. Difficulties in maintaining diet and exercise programs (58.8%, n=190) and insufficient weight loss (50.2%, n=162) were the main cited reasons for discontinuing weight loss efforts. Overall, 68.2% (n=217) reported dissatisfaction with their current weight and believed further weight loss was possible. Consistent with this, 60.6% (n=197) reported feeling highly or very bothered by their weight, and 39.2% (n=128) indicated embarrassment in public because of it (Table 4).

**Table 4:** PwO objectives for losing weight and feelings toward weight. PwO reported data. *The labels for options 2, 3, and 4 were added retrospectively (i.e., after data collection) to aid data interpretation. Abbreviation: PwO, people with overweight/obesity.

Question	N (%)
Objectives for losing weight among those not reaching goal weight; N available	296
To look better (physical appearance)	196 (66.2)
To feel better about myself	182 (61.5)
To fit into smaller clothes	163 (55.1)
To feel more confident	157 (53.0)
To be able to exercise regularly	152 (51.4)
To feel less physical discomfort	149 (50.3)
To reduce my joint pain	140 (47.3)
To be able to do more around the home	125 (42.2)
To feel less anxious/depressed	111 (37.5)
My sleeping improves	110 (37.2)
I am out of breath less often	81 (27.4)
To be able to get outside more	75 (25.3)
I have no expectations	9 (3.0)
Other	4 (1.4)
Objectives for losing weight among those reaching goal weight; N available	233
To look better (physical appearance)	114 (48.9)
To feel better about myself	108 (46.4)
To fit into smaller clothes	104 (44.6)
To feel more confident	81 (34.8)
To reduce my joint pain	79 (33.9)
To be able to do more around the home	75 (32.2)
My sleeping improves	75 (32.2)
To feel less physical discomfort	73 (31.3)
I am out of breath less often	71 (30.5)
To be able to exercise regularly	68 (29.2)
To be able to get outside more	49 (21.0)
To feel less anxious/depressed	44 (18.9)
I have no expectations	12 (5.2)
Other	3 (1.3)
Weight loss goal has been reached; N available	322
No	239 (74.2)
I do not have a weight loss goal/target	51 (15.8)
Yes	32 (9.9)
Extend to lifestyle changes implemented; N available	323
1- No changes	26 (8.1)
2- Minimal changes*	61 (18.9)
3- Moderate changes*	119 (36.8)
4- Substantial changes*	90 (27.9)
5- Totally changed lifestyle	27 (8.4)
PwO feelings to diet/exercise program; N available	319
I do my best to follow it but sometimes don't succeed	231 (72.4)
I only follow the diet programme	36 (11.3)
I follow it completely	25 (7.8)
I only follow the exercise programme	15 (4.7)
I don't follow it at all	12 (3.8)
Reasons why stopped trying to lose weight; N available	323
When I find my diet and exercise programme too hard to keep going	190 (58.8)
I give up when I am not losing enough weight	162 (50.2)
When I feel emotional (for example, when I am upset, anxious or depressed)	109 (33.7)
I have never stopped trying to lose weight	26 (8.0)
When I have lost enough weight	20 (6.2)
When I get side effects from the medicine, I take to help me lose weight	12 (3.7)
Other	11 (3.4)
How happy PwO are about current weight; N available	318
I am not happy with it, and I think I can lose more weight	217 (68.2)
I am happy with it even though I have not reached my goal/target weight	52 (16.4)
I am not happy with it, but I think it is the best that I can achieve	41 (12.9)
I am happy with it, and I feel I have reached my goal/target weight	8 (2.5)
How bothered PwO are about weight; N available	325
1- Not at all bothered	11 (3.4)
2- Slightly bothered*	26 (8.0)
3- Moderately bothered*	91 (28.0)
4- Highly bothered*	124 (38.2)
5- Very bothered	73 (22.5)
PwO embarrassment level regarding weight in public; N available	327
1- I am not at all embarrassed	55 (16.8)
2- I sometimes feel embarrassed*	61 (18.7)
3- I often feel embarrassed*	83 (25.4)
4- I frequently feel embarrassed*	84 (25.7)
5- I am always embarrassed	44 (13.5)

Impact of obesity on HRQoL and work-related outcomes

**Figure 1:**
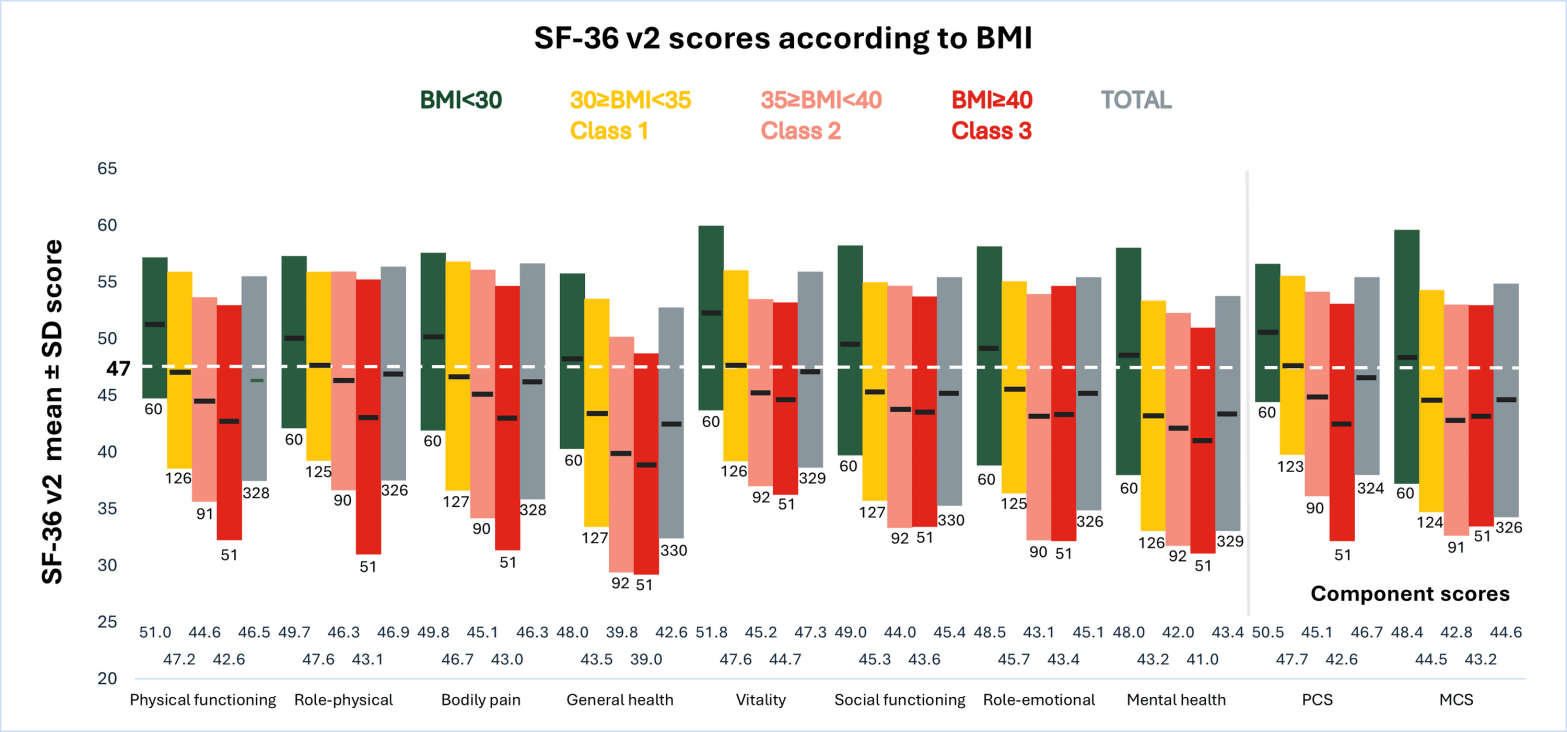
SF-36v2 scores according to BMI value Values ranged from 0 to 100, with higher values indicating better HRQoL and values <47 indicating deterioration (dashed line). N below each bar indicates the number of cases. Abbreviations: BMI, body mass index; MCS, mental component summary; PCS, physical component summary; SF-36v2, 36-Item Short Form Health Survey version 2.

The mean SF-36v2 scores were below 47 for all domains and component summary scores evaluated, except for vitality (47.3), indicating PwO experienced deterioration across multiple dimensions. Overall, the greatest HRQoL impairment was found in PwO with BMI ≥40 kg/m², particularly affecting general health (39.0 ± 9.8), mental health (41.0 ± 10.0), and physical functioning (42.6 ± 10.4) domains. In contrast, participants with BMI <30 kg/m² showed the highest scores, especially in vitality (51.8 ± 8.1), physical functioning (51.0 ± 6.2), and general health (48.0 ± 7.7) (Figure 1). SF-36v2 scores by EOSS stage are shown in the Appendices (Supplemental Material 4).

**Figure 2:**
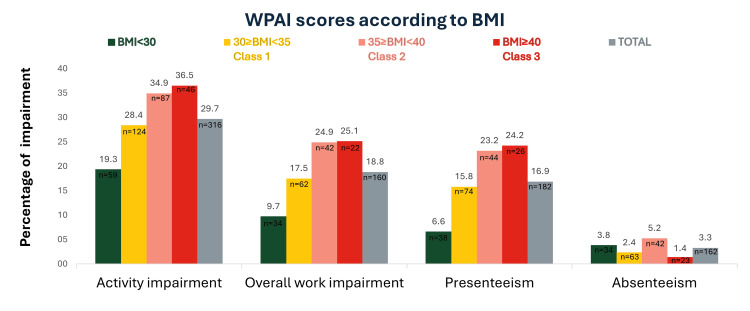
WPAI scores according to BMI value Percentages show the extent to which work and daily activities are affected by overweight/obesity (0% = no impairment; 100% = activity completely impaired). Abbreviations: BMI, body mass index; WPAI, work productivity and activity impairment.

Among individuals with a BMI <30 kg/m², 65.5% (n=38) were working, and this percentage decreased to 60.4% (n=29) among those with class III obesity. A progressive increase in proportions of non‑employment was observed across ascending BMI categories (Table 2). Among employed PwO, work impairment due to overweight/obesity was 18.8%, while activity impairment, regardless of employment status, was 29.7% (Figure 2). PwO with BMI ≥40 kg/m² reported the highest WPAI scores, with activity impairment at 36.5%, overall work impairment at 25.1%, and presenteeism at 24.2%. WPAI scores by EOSS stage are shown in the Appendices (Supplemental Material 5).

PwO and physicians' perceived reasons for obesity

**Figure 3:**
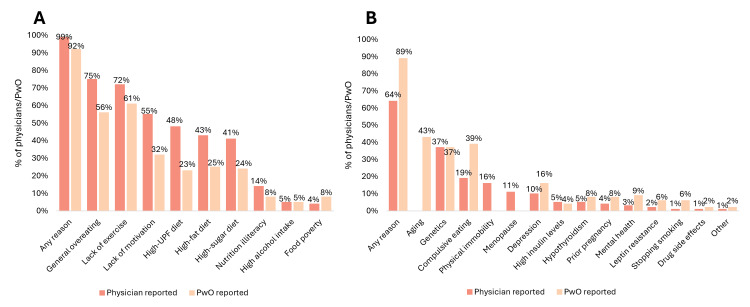
PwO and physicians' perceived reasons for obesity (A) Behavioral/socioeconomic reasons. (B) Biological reasons. Abbreviations: PwO, people with overweight/obesity. UPF, ultra-processed food.

Both physicians and PwO identified a lack of exercise and general overeating as the primary contributors to overweight/obesity. However, perspectives differed regarding other contributing factors. Physicians cited a lack of PwO motivation as the third most common reason, followed by dietary factors such as the consumption of highly processed foods and diets high in sugar or fat. In contrast, PwO placed less emphasis on diet-related reasons and instead attributed their weight gain to aging and difficulty controlling their eating habits (Figures 3A, 3B).

## Discussion

In this cross-sectional real-world study of physician- and PwO-reported data, overweight and obesity were accompanied by several clinical, humanistic, and economic outcomes that may broadly and negatively affect the lives of people living with this disease.

Excess weight might be linked to a high comorbidity burden, since 88% of PwO presented at least one ORD. Individuals with obesity Class II or III had a higher average number of complications, emphasizing the progressive nature of obesity-related health deterioration [30,31]. Furthermore, the mean BMI at the time of diagnosis was 37.4 kg/m² (Class II Obesity). This suggests that many PwO are being diagnosed at a relatively advanced stage, which could be indicative of underdiagnosis or delayed intervention. Additionally, individuals with obesity class III had the lowest mean age, underscoring that obesity is increasingly affecting younger populations. This has substantial implications for long-term health outcomes and productivity across the life course [32].

Findings from the SF-36v2 questionnaire suggest that increasing BMI is associated with declining HRQoL. People included in the study face a significant burden of comorbidities, and their presence has been documented to affect both physical and general health [21].

In the psychosocial domain, PwO reported poorer quality of life and an increased risk of certain psychiatric disorders, most notably depression. A long-term study found PwO to have lower HRQoL and life satisfaction compared with individuals of a normal weight [13,33]. Furthermore, a recent meta-analysis by Luppino et al. [34] detailed the relationship between obesity and depression and noted the potentially reciprocal nature of this association, with obesity increasing the risk of depression and depression being predictive of gaining significant weight. The emotional and psychological dimensions of overweight/obesity are often overlooked, despite their substantial impact on PwO’s ability to sustain lifestyle changes and achieve long-term goals [35]. Taken together and given that only 10% of PwO were receiving psychological support at the time of data collection, these results reinforce the need for comprehensive obesity management strategies aimed at enhancing physical and mental well-being.

In parallel, data from the WPAI questionnaire suggested an effect of high BMI on work productivity. Work impairment and the impact of presenteeism showed an upward trend with increasing BMI, potentially contributing to higher indirect costs. Indeed, economic analyses suggest that obesity may contribute substantially to both direct and indirect healthcare expenditures.

The study findings provide compelling evidence of an unmet clinical need among PwO. Despite the known benefits of weight reduction, the study reveals a modest average decrease in BMI from diagnosis to assessment (-2.2 kg/m²). More than half of the PwO experienced weight regain after initial weight loss, and more than one-tenth reported never achieving any weight loss at all. Furthermore, adherence remains a significant challenge since, in our sample, more than half of PwO discontinued their weight loss programs, citing difficulty adhering to plans and frustration due to a lack of perceived progress. Notably, most participants were managed exclusively with lifestyle-based strategies (dietary counselling, physical-activity advice, and behavioral therapy). Although such measures are foundational, they generally yield only modest absolute reductions, and their effects tend to plateau within the first 6-12 months [36]. Moreover, sustained caloric restriction triggers powerful counter-regulatory physiology. Adaptive thermogenesis reduces resting energy expenditure, while orexigenic hormonal shifts heighten subjective appetite [37]. These mechanisms collectively drive a biological imperative to regain lost weight, further complicating long-term adherence and highlighting the limitations of lifestyle intervention as a stand-alone therapy.

In our sample, the utilization of OMM and bariatric surgery was low, even though both are recognized as therapeutic strategies that achieve greater reductions in body weight [11]. In Spain, these limitations could be attributed to the fact that the Spanish National Health System (SNS) excludes OMM from public funding. Moreover, although bariatric surgery is recommended for adults aged 18-60 years with Class I or Class II Obesity with associated severe comorbidities [11], access is hampered by protracted waiting lists and the risk of post-surgery complications [38-40]. The obesity management practices described in this study differ from recommendations in the GIRO guideline [11], suggesting a potential implementation gap and indicating that routine clinical practice may not fully align with evidence-based, guideline-directed, comprehensive obesity care [33,41].

Our study possesses some strengths, including providing new insights into the humanistic impact of obesity in Spain using standardized research methodology across a substantial sample of physicians and PwO. However, some limitations inherent to the data source and study design apply. First, the study is subject to potential selection bias at both the physician and patient levels. Participating physicians were drawn from a voluntary panel, which may not fully reflect the broader population of HCPs in Spain. Similarly, as participation in patient‑reported components was voluntary and required digital access, individuals from lower socioeconomic backgrounds or those more likely to be digitally excluded (e.g., older adults or people with limited internet access) may be under‑represented. Additionally, missing data were not imputed, and since non‑respondents may differ from respondents, the magnitude and direction of any resulting bias cannot be determined. Consequently, results should be interpreted with caution, as they may not be generalizable to all HCPs managing obesity and consulting PwO in Spain. Second, the cross‑sectional nature of the study does not allow the evaluation of longitudinal changes in patient-reported outcomes over time, so it is not possible to determine whether these outcomes improve, worsen, or fluctuate. Third, no objective measures of work productivity or activity impairment were available, as outcomes relied solely on self‑reported instruments. Reliance on self‑reported measures may introduce recall or perception bias, and the lack of corroborating administrative or device‑based data potentially limits interpretation of the true magnitude of impairment.

## Conclusions

In conclusion, our study revealed that most of the PwO in the sample exhibited clinical complications related to excess weight. Additionally, overweight and obesity negatively affected all dimensions of HRQoL on daily activities and work productivity, and this burden was evident among individuals with elevated BMI and/or EOSS. These findings underscore the need for comprehensive, personalized care strategies that address both the physical and emotional dimensions of obesity. By highlighting the real-world burden of obesity, this study may help guide the development of person-centered treatment approaches aimed at improving disease management and long-term health outcomes.

## Figures and Tables

**Figure 4:**
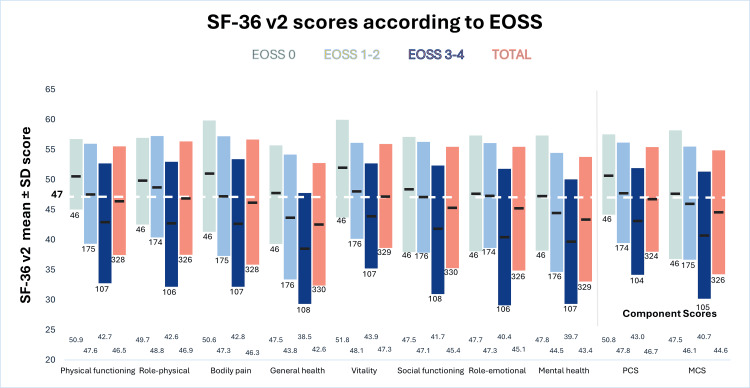
SF-36v2 scores by EOSS stages Values ranged from 0 to 100, with higher values indicating better HRQoL and values <47 indicating deterioration (dashed line). N below each bar indicates the number of cases. Abbreviations: Edmonton Obesity Staging System (EOSS); MCS, mental component summary; PCS, physical component summary; SF-36v2, 36-Item Short Form Health Survey version 2.

**Figure 5:**
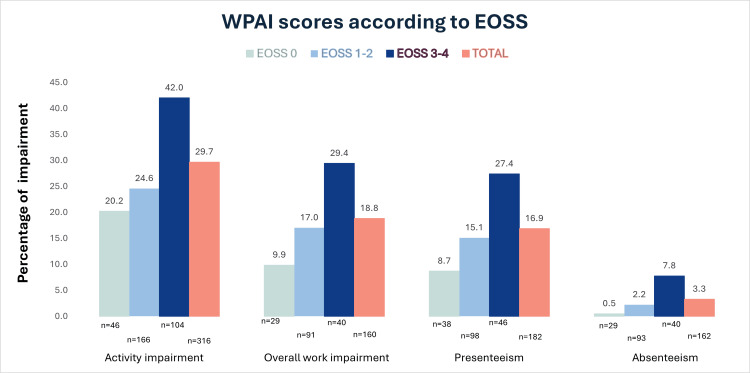
WPAI scores by EOSS stages Percentages show the extent to which work and daily activities are affected by overweight/obesity (0% = no impairment; 100% = activity completely impaired). Abbreviations: Edmonton Obesity Staging System (EOSS); WPAI, Work Productivity and Activity Impairment.

## Appendices

Supplemental material 1

**Table 5:** Adelphi EOSS criteria definition Abbreviations: EOSS, Edmonton Obesity Staging System; T2DM, type 2 diabetes mellitus; PwO, people with overweight/obesity; CV, cardiovascular disease.

EOSS category	DSP definition
EOSS 0	Physician selected ‘Not diagnosed with any concomitant conditions’ or none of any concomitant conditions listed under EOSS 1-2 or EOSS 3-4. AND Physician did not select ‘Compulsive eating disorder/food addiction’ AND Physician selected ‘No additional support/care’
EOSS 1-2	At least 1 of the following concomitant conditions/functional limitations was selected by the physician: Compulsive eating disorder/food addiction Hypertension Dyslipidemia Prediabetes T2DM without chronic complications NAFLD or NASH AND stage F2 according to liver biopsy Lumbago Gout Gastroesophageal Reflux Disease Sleep apnoea Asthma Tiredness Moderate depression depression/anxiety (defined as depression present and not receiving anti-depressant drugs or Anxiety present and not receiving anti-anxiety drugs/benzodiazepines) Anxiety Moderate functional limitations, physician selected that the PwO needs help with any of the following: Transportation: Being able to attend events. Being able to manage transportation, either via driving or by organizing other means of transport. Managing finances: Being able to pay bills and manage financial assets. Shopping and meal preparation: Everything required to get a meal on the table and shopping for clothing and other items required for daily life. Housecleaning and home maintenance: Cleaning after eating, maintaining clean and tidy living areas, and keeping up with home maintenance. Managing communication with others: Being able to manage calls, messaging and mail. And none of the following concomitant conditions/functional limitations was selected by the physician: CV disease: MI Stroke Heart failure T2DM with chronic complications Diabetic ketoacidosis Diabetic neuropathic pain Renal disease Polycystic ovary syndrome Acute respiratory distress syndrome Severe depression/anxiety (defined as depression present and receiving anti-depressant drugs or Anxiety present and receiving anti-anxiety drugs/benzodiazepines) Severe functional limitations, physician selected the PwO did not need help with any of the following: Ambulating: Being able to move from one position to another and walk about Feeding: Being able to feed themself Dressing: Being able to select appropriate clothes and to put the clothes on Personal hygiene: Being able to wash and groom themself and maintain hygiene Continence: Cleaning due to a loss of control of bladder or bowel function Toileting: Being able to get to and from the toilet, using it appropriately, and cleaning themselves Managing medications: Being able to obtain medications and take them as directed.
EOSS 3-4	At least 1 of the following concomitant conditions/functional limitations was selected by the physician: CV disease: MI Stroke Heart failure T2DM with chronic complications Diabetic ketoacidosis Diabetic neuropathic pain Renal disease Polycystic ovary syndrome Osteoarthritis Acute respiratory distress Severe depression/anxiety (defined as depression present and receiving anti-depressant drugs or Anxiety present and receiving anti-anxiety drugs/benzodiazepines) Severe functional limitations, physician selected that the PwO needs help with any of the following: Ambulating: Being able to move from one position to another and walk about Feeding: Being able to feed themself Dressing: Being able to select appropriate clothes and to put the clothes on Personal hygiene: Being able to wash and groom themself and maintain hygiene Continence: Cleaning due to a loss of control of bladder or bowel function Toileting: Being able to get to and from the toilet, using it appropriately, and cleaning themselves Managing medications: Being able to obtain medications and take them as directed.

Supplemental material 2

**Table 6:** Demographic and clinical characteristics of PwO at the time of the survey by EOSS stages ^ Includes: “full-time homemaker”, “student”, “unemployed”, and “on long-term sick”. ^^ Includes “completed mandatory education”, “did not complete mandatory education”, and “other”. *Values with N≤3 have been suppressed to protect confidentiality. Additional cells may also have been suppressed to prevent the deduction of small counts. **Those present in >10% of participants were included. Abbreviations: ACE, angiotensin-converting enzyme; ARB, angiotensin-II receptor; BMI, body mass index; EOSS, Edmonton Obesity Staging System; PwO, person with obesity; PwOW, person with overweight; SD, standard deviation; SGLT-2, sodium-glucose cotransporter 2; T2DM, type 2 diabetes mellitus.

Variables	Total; n=801 (100%)	EOSS 0; n=125 (15.6%)	EOSS 1-2; n=392 (48.9%)	EOSS 3-4; n=284 (35.5%)
Age, mean (SD)	51.0 (13.7)	44.1 (11.5)	50.9 (12.5)	54.3 (15.1)
Sex, women; n (%)	511 (63.8)	81 (64.8)	241 (61.5)	189 (66.5)
BMI at diagnosis, mean (SD)	37.4 (6.8)	37.1 (9.1)	37.2 (6.5)	37.8 (6.0)
BMI at data collection; mean (SD)	35.4 (6.5)	34.3 (8.5)	35.2 (6.2)	36.3 (5.7)
Time from diagnosis, years; mean (SD)	5.0 (6.3)	3.6 (4.6)	4.9 (6.1)	5.8 (7.0)
Smoking status, n	778	123	377	278
Never smoked, n (%)	462 (59.4)	84 (68.3)	220 (58.4)	158 (56.8)
Ex-smoker, n (%)	201 (25.8)	19 (15.4)	99 (26.3)	83 (29.9)
Current smoker, n (%)	115 (14.8)	20 (16.3)	58 (15.4)	37 (13.3)
Employment status, n	320	46	169	105
Working, n (%)	196 (61.3)	39 (84.8)	110 (65.1)	47 (44.8)
Not working^, n (%)	60 (18.8)	… (*)	… (*)	22 (21.0)
Retired, n (%)	64 (20.0)	… (*)	… (*)	36 (34.3)
Educational status, n	327	45	175	107
University degree or higher, n (%)	114 (34.9)	25 (55.6)	63 (36.0)	26 (24.3)
Completed non-mandatory secondary education, n (%)	60 (18.3)	9 (20.0)	35 (20.0)	16 (15.0)
Mandatory education or lower^^, n (%)	153 (46.8)	11 (24.4)	77 (44.0)	65 (60.7)
Obesity-related diseases, mean (SD)	2.5 (2.2)	0.4 (0.6)	2.2 (1.5)	3.9 (2.5)
Obesity-related diseases, n (%)				
Any obesity-related disease**	707 (88.3)	46 (36.8)	378 (96.4)	283 (99.6)
Hypertension	330 (41.2)	0 (0)	193 (49.2)	137 (48.2)
Dyslipidemia	263 (32.8)	0 (0)	159 (40.6)	104 (36.6)
Anxiety	151 (18.9)	0 (0)	50 (12.8)	101 (35.6)
T2DM without chronic complications	112 (14.0)	0 (0)	75 (19.1)	37 (13.0)
Depression	110 (13.7)	0 (0)	6 (1.5)	104 (36.6)
Osteoarthritis	99 (12.4)	0 (0)	0 (0)	99 (34.9)
Lumbago	96 (12.0)	0 (0)	43 (11.0)	53 (18.7)
Hypothyroidism	90 (11.2)	12 (9.6)	42 (10.7)	36 (12.7)
Concomitant medication use, n	707	46	378	283
Concomitant medications**, n (%)				
Any concomitant medication	658 (93.1)	33 (71.7)	353 (93.4)	272 (96.1)
Statin	274 (38.8)	8 (17.4)	152 (40.2)	114 (40.3)
ARB inhibitors	202 (28.6)	3 (6.5)	118 (31.2)	81 (28.6)
Anti-depressants	172 (24.3)	3 (6.5)	22 (5.8)	147 (51.9)
Metformin/Biguanide	155 (21.9)	3 (6.5)	78 (20.6)	74 (26.1)
Benzodiazepines	148 (20.9)	1 (2.2)	62 (16.4)	85 (30.0)
ACE inhibitors	119 (16.8)	1 (2.2)	61 (16.1)	57 (20.1)
Pain medication	112 (15.8)	2 (4.3)	45 (11.9)	65 (23.0)
Other diurectics	92 (13.0)	0 (0)	44 (11.6)	48 (17.0)
SGLT-2 inhibitors	86 (12.2)	2 (4.3)	40 (10.6)	44 (15.5)
Calcium channel blockers	84 (11.9)	0 (0)	40 (10.6)	44 (15.5)

Supplemental material 3

**Table 7:** Overview of bariatric surgery utilization and eligibility *Other includes “Laparoscopic adjustable gastric banding”, “Duodenal switch with biliopancreatic division”, and “Other” categories.

Question	N (%)
Use of weight loss surgery as part of current and previous weight loss attempts	
N available	801
Weight loss surgery was used as part of a previous weight loss attempt	22 (2.7)
Weight loss surgery is being used as part of their current weight loss attempt	28 (3.5)
Type of bariatric surgery received	
N available	46
Total number of patients who received at least one weight loss surgery	34 (73.9)
Roux-en-Y gastric bypass	14 (30.4)
Sleeve gastrectomy	10 (21.7)
Other*	13 (28.3)
Don't know	12 (26.1)
Candidacy for weight loss surgery	
N available	801
Yes, candidate for weight loss surgery	211 (26.3)
No, not a candidate for weight loss surgery	407 (50.8)
Don't know	156 (19.5)
No response	27 (3.4)
Analysis on candidacy for surgery, and whether they have received surgery	
N available	632
Received bariatric surgery	32 (5.1)
Candidate for bariatric surgery, but not received bariatric surgery	196 (31.0)
Not a candidate for bariatric surgery, not received bariatric surgery	404 (63.9)
Reasons for not being a candidate for weight loss surgery	
N available	407
Patient weight/risk assessment is below weight loss surgery recommendation guidelines	189 (46.4)
All appropriate non-surgical measures have not yet been exhausted	174 (42.8)
I do not feel this patient will commit to the long-term follow-up requirements	66 (16.2)
This patient is not fit for anaesthesia or surgery	26 (6.4)
I have reservations for this patient around safety of procedure	26 (6.4)
The patient's mental health prevents them qualifying for weight loss surgery	22 (5.4)
Other	22 (5.4)
The cost is prohibitive to offering weight loss surgery to this patient	9 (2.2)
Don't know	5 (1.2)

Supplemental material 4

Supplemental material 5
